# Determination of triacylglycerol oxidation mechanisms in canola oil using liquid chromatography–tandem mass spectrometry

**DOI:** 10.1038/s41538-017-0009-x

**Published:** 2018-01-12

**Authors:** Shunji Kato, Naoki Shimizu, Yasuhiko Hanzawa, Yurika Otoki, Junya Ito, Fumiko Kimura, Susumu Takekoshi, Masayoshi Sakaino, Takashi Sano, Takahiro Eitsuka, Teruo Miyazawa, Kiyotaka Nakagawa

**Affiliations:** 1 Food and Biodynamic Chemistry Laboratory, Graduate School of Agricultural Science, Tohoku University, Sendai, Miyagi 980-0845 Japan; 2 Department of Cell Biology, Division of Host Defense Mechanism, Tokai University School of Medicine, Isehara, Kanagawa 259-1193 Japan; 3 Department of Human Health and Nutrition, Shokei Gakuin University, Natori, Miyagi 981-1295 Japan; 4 Fundamental Research Laboratory, J-OIL MILLS, INC., Yokohama, Kanagawa 230-0053 Japan; 5 Food and Biotechnology Innovation Project, New Industry Creation Hatchery Center (NICHe), Tohoku University, Sendai, Miyagi 980-8579 Japan; 6 Food and Health Science Research Unit, Graduate School of Agricultural Science, Tohoku University, Sendai, Miyagi 981-8555 Japan

**Keywords:** Industry, Technology

## Abstract

Triacylglycerol (TG), the main component of edible oil, is oxidized by thermal- or photo- oxidation to form TG hydroperoxide (TGOOH) as the primary oxidation product. Since TGOOH and its subsequent oxidation products cause not only the deterioration of oil quality but also various toxicities, preventing the oxidation of edible oils is essential. Therefore understanding oxidation mechanisms that cause the formation of TGOOH is necessary. Since isomeric information of lipid hydroperoxide provides insights about oil oxidation mechanisms, we focused on dioleoyl-(hydroperoxy octadecadienoyl)-TG (OO-HpODE-TG) isomers, which are the primary oxidation products of the most abundant TG molecular species (dioleoyl-linoleoyl-TG) in canola oil. To secure highly selective and sensitive analysis, authentic OO-HpODE-TG isomer references (i.e., hydroperoxide positional/geometrical isomers) were synthesized and analyzed with HPLC-MS/MS. With the use of the method, photo- or thermal- oxidized edible oils were analyzed. While dioleoyl-(10-hydroperoxy-8*E*,12*Z*-octadecadienoyl)-TG (OO-(10-HpODE)-TG) and dioleoyl-(12-hydroperoxy-9*Z*,13*E*-octadecadienoyl)-TG (OO-(12-HpODE)-TG) were characteristically detected in photo-oxidized oils, dioleoyl-(9-hydroperoxy-10*E*,12*E*-octadecadienoyl)-TG and dioleoyl-(13-hydroperoxy-9*E*,11*E*-octadecadienoyl)-TG were found to increase depending on temperature in thermal-oxidized oils. These results prove that our methods not only evaluate oil oxidation in levels that are unquantifiable with peroxide value, but also allows for the determination of oil oxidation mechanisms. From the analysis of marketed canola oils, photo-oxidized products (i.e., OO-(10-HpODE)-TG and OO-(12-HpODE)-TG) were characteristically accumulated compared to the oil analyzed immediately after production. The method described in this paper is valuable in the understanding of oil and food oxidation mechanisms, and may be applied to the development of preventive methods against food deterioration.

## Food chemistry: new method probes how oils go rancid

Edible oils become rancid when reacting with oxygen under light or heat, degrading into different products depending on the pathway. Kiyotaka Nakagawa at Tohoku University, Japan, and co-workers used instruments that can separate and identify by weight components in mixtures to study light- and heat-induced oxidation of canola oil. Using authentic samples of possible oxidation products as references, the team found that each process generated two unique species from triacylglycerol, the main ingredient in edible oils. These signature compounds allowed the researchers to reveal that heat-oxidation sped up as temperature increased and that light-oxidized products gradually accumulated in off-the-shelf canola oil after production. This method is more sensitive than conventional protocols and can tell exactly how oils are oxidized, useful for developing techniques for food preservation.

## Introduction

**Fig. 1:**
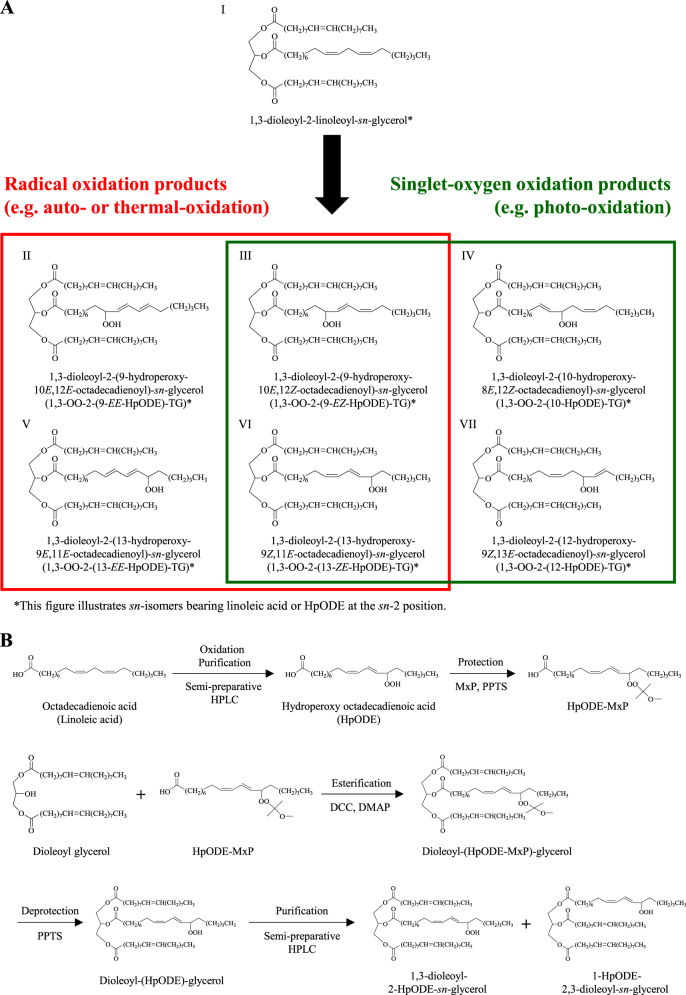
Triacylglycerol (TG) oxidation mechanisms and the structures of triacylglycerol hydroperoxide isomers (**a**). Dioleoyl-linoleoyl glycerol (I) is oxidized to dioleoyl-(hydroperoxy octadecadienoyl) glycerol (OO-HpODE-TG, II–VII) by radical and/or singlet-oxygen oxidation. Hydroperoxide positions and geometrical structures depend on the TG peroxidation mechanisms. Schemes for the reference preparation of OO-HpODE-TG isomers (**b**). The references were synthesized from dioleoyl glycerol and HpODE isomers

Edible oil is an essential food ingredient that is mainly composed of triacylglycerol (TG), and is widely used in various food products. During industrial or culinary processes, TG oxidizes to TG hydroperoxide (TGOOH) as the primary oxidation product by radical (e.g., auto-, thermal-) oxidation or singlet-oxygen (e.g., photo-) oxidation (Fig. 1a),^1,2^ and subsequently oxidized to various secondary oxidation products (e.g., aldehydes and carboxylates).^1–3^ The sequential formation of these oxidative products not only deteriorate the oil quality such as nutritional value, flavor and taste, but also exhibit various toxicities.^4–8^ Staprans et al. reported that dietary oxidized lipids are absorbed by the small intestine and distributed to lipoproteins and the liver.^9–11^ The circulating (and/or accumulated) oxidized lipids are presumed to play an important role in the development of various disorders such as cardiovascular disease,^12–14^ Alzheimer’s disease,^15^ and aging.^16,17^ Therefore, the prevention of TGOOH generation in oil is beneficial to our health and key to maintain the quality of edible oils. Based on these facts, considerable attention has been paid on how to analyze TGOOH and to determine the cause of TGOOH generation.^18–21^

Classically, TGOOH is quantified by volumetric (e.g., peroxide value (POV))^22^ or spectroscopic (e.g., ferrous oxidation xylenol orange (FOX))^23^ methods. Of these methods, POV has been certified as an official method by the Association of Official Agricultural Chemists (AOAC),^22^ because of its inexpensiveness and convenience. However, since these methods are based on the oxidation–reduction reaction of iodine or ferrous, there are some drawbacks such as selectivity, sensitivity and interference of matrix compounds. To overcome such drawbacks, some selective and sensitive chromatographic methods were reported during the past few decades.^24–30^ For example, with the use of reverse phase (RP) chemiluminescence (CL) HPLC, we detected mono-, bis- and tris- hydroperoxides of certain TG molecular species such as trilinoleoyl-glycerol and oleoyl-dilinoleoyl-glycerol.^25^ Also, Alam et al. identified mono- and bis- hydroperoxides of trioleoyl-glycerol in olive oil using RP-HPLC coupled with electrospray-ionization mass spectrometry (ESI-MS).^28^ However, these previous methods were unable to provide information about the geometrical and positional isomers of the fatty acid hydroperoxide in TG, despite the fact that these isomeric information is helpful in the elucidation of TG oxidation mechanisms (i.e., auto-, thermal- or photo-oxidation) (Fig. 1).^1,2^ Recently, we found that the use of alkali metals (e.g., sodium) on ESI tandem mass spectrometry (MS/MS) enables analysis of lipid hydroperoxide positional isomers,^31–37^ and with the use of this technique, it was identified that the oxidation of phospholipids in mayonnaise was predominantly initiated by auto-oxidation.^37^

In this study, we analyzed TGOOH isomers in canola oil, an edible oil commonly used worldwide. The aim of this study was to analyze TGOOH high-sensitively, and to determine the oil oxidation mechanisms. Because the analysis of TGOOH is complex due to the many factors being involved (i.e., the type and position of fatty acids on the glycerol backbone, and the positional/geometric isomerism of the hydroperoxide group), we first determined the most suitable TGOOH molecular species for the evaluation of canola oil oxidation mechanisms. Authentic references of the target TGOOH isomers were then prepared. Using the references, we quantified TGOOH isomers in canola oil by HPLC–ESI-MS/MS. Information on the geometrical and hydroperoxide positional isomers of TGOOH gave an insight into the TGOOH generation mechanisms in canola oil. The method described in this paper is valuable not only in the understanding of the cause of food oxidation during industrial or culinary processes, but also in developing preventive methods, including the choice of suitable anti-oxidants and packaging techniques depending on the identified oxidation mechanisms.

## Result and discussion

### Determination of target TGOOH for the evaluation of oil oxidation mechanisms

Edible oil is composed of various TG molecular species. Since certain fatty acids generate multiple fatty acid hydroperoxide isomers (e.g., two types of isomers from oleic acid (OA) or six types of isomers from linoleic acid (LA)),^1,2,33^ it can be assumed that incalculable types of TGOOH molecular species/isomers are contained in oxidized edible oil. And this is the reason why the determination/quantification of edible oil oxidation is very difficult.^29^ Hence, we first determined the most suitable TGOOH molecular species for the evaluation of canola oil oxidation mechanisms. Based on GC analysis, it was identified that the main fatty acids that compose canola oil TG were OA (59.7%), LA (20.0%), and linolenic acid (LnA) (8.9%) (Data not shown). The fatty acid combinations of TG were then determined by HPLC-MS operated in the Q1 mass- and product ion-scan mode. In the Q1 mass chromatogram, *m/z* 899.8, *m/z* 901.9, *m/z* 903.9, *m/z* 906.0, and *m/z* 907.9 were abundantly detected (SI 1A). To analyze the fatty acid composition of these TG molecular species, product ion scan was performed (SI 1B). From the product ion analysis, we found that *m/z* 899.8, *m/z* 901.9, and *m/z* 903.9 were composed of at least two types of TG molecular species. Collision induced dissociation (CID) of peak I (left side of *m/z* 899.8) produced *m/z* 620.6 and *m/z* 622.8, corresponding to the desorption of either a LA or LnA residue, respectively (SI 1C). In consideration of monoisotopic mass (*m/z* 899.7: [M+Na]^+^), *m/z* 899.8 (left side) was considered to be dilinoleoyl-linolenyl glycerol. On the other hand, CID of peak II (right side of *m/z* 899.8) produced *m/z* 618.7 and *m/z* 622.8 (SI 1D), corresponding to the desorption of either an OA or LnA residue, respectively. Based on these results, the right side of *m/z* 899.8 was then considered to be oleoyl-dilinolenyl glycerol. Also, CID of peak III produced only *m/z* 622.8, which identified peak III as trilinoleoyl glycerol (SI 1E). Similarly, peaks IV–VIII were identified as oleoyl-linoleoyl-linolenyl glycerol (SI 1F), oleoyl-dilinoleoyl glycerol (SI 1G), dioleoyl-linolenyl glycerol (SI 1H), dioleoyl-linoleoyl glycerol (SI 1I), and trioleoyl glycerol (SI 1J), respectively.

Of these TG, we considered that the most suitable TG molecular species for the evaluation of oxidation mechanisms are the ones that generate different isomers corresponding to different oxidation mechanisms. OA is hardly oxidized by thermal-oxidation,^38^ because thermal-oxidation (i.e., radical oxidation) is initiated by the abstraction of a hydrogen atom from the bis-allylic site, which OA does not posess.^1,2,30^ On the other hand, because photo-oxidation (i.e., singlet oxygen-oxidation) directly occurs at double bonds by the so-called “ene” reaction,^1^ peroxidation of polyunsaturated fatty acid (PUFA) such as LnA generates excessive types of hydroperoxide isomers.^1,2,30^ Based on these results and facts, we considered that dioleoyl-(hydroperoxy octadecadienoyl) glycerol (OO-HpODE-TG), which possesses one molecule of LA hydroperoxide (hydroperoxy octadecadienoic acid: HpODE), is the most suitable molecular species for the evaluation of canola oil oxidation mechanisms. Because there are six types of HpODE isomers (i.e., 9-hydroperoxy-10*E*,12*Z*-octadecadienoic acid: 9-*EZ*-HpODE, 9-hydroperoxy-10*E*,12*E*-octadecadienoic acid: 9-*EE*-HpODE, 10-hydroperoxy-8*E*,12*Z*-octadecadienoic acid: 10-HpODE, 12-hydroperoxy-9*Z*,13*E*-octadecadienoic acid: 12-HpODE, 13-hydroperoxy-9*Z*,11*E*-octadecadienoic acid: 13-*ZE*-HpODE and 13-hydroperoxy-9*E*,11*E*-octadecadienoic acid: 13-*EE*-HpODE),^33^ in this study, we analyzed each OO-HpODE-TG isomer including their *sn*-isomers (i.e., OO-HpODE-TG bearing HpODE isomers at *sn*-1 and *sn*-2 positions).

### Preparation of OO-HpODE-TG isomer references

In a previous study, Alam analyzed oxidized camellia oil using HPLC–ESI-MS.^29^ However, the study only provided the “possible” identification of TG auto-oxidation products, because the analysis depended solely on *m/z* values and did not apply any references. Since there are numerous TGOOH molecular species/isomers in oxidized TG, such lack of authentic references may cause fallacious analysis. In this study, to achieve highly selective analysis of TGOOH molecular species/isomers, we synthesized OO-HpODE-TG isomer references from diacylglycerol and six HpODE isomers (Fig. 1b). Since it is difficult to chromatographically separate esterified HpODE isomers,^31^ the six HpODE isomers were first separated and collected in its free form, and subsequently each esterified with dioleoyl glycerol.^39^ Because we used a mixture of dioleoyl glycerol isomers (i.e., 1,3-dioleoyl-2-hydroxy-*sn*-glycerol and 1-hydroxy-2,3-dioleoyl-*sn*-glycerol), the obtained OO-HpODE-TG was a mixture of 1,3-dioleoyl-2-(HpODE)-*sn*-glycerol (1,3-OO-2-(HpODE)-TG) and 1-(HpODE)-2,3-dioleoyl-*sn*-glycerol (1-(HpODE)-2,3-OO-TG). But, even when pure 1,2-dioleoyl-3-hydroxy-*sn*-glycerol is employed, the resultant OO-HpODE-TG is known to be a mixture of the isomers, because 1,2-diacyl glycerol isomerizes to 1,3-diacyl glycerol which is thermodynamically more stable.^40^ Hence, to avoid this isomerization reaction, in this study, OO-HpODE-TG *sn*-isomers were separated after esterification. In order to determine the HpODE binding *sn*-position of the obtained OO-HpODE-TG, a portion of the references were catalyzed by α-lipase, which act specifically at the *sn*-1 and *sn*-3 positions of triacylglycerol. Before this reaction, the hydroperoxide group of OO-HpODE-TG was protected with 2-methoxypropene (MxP)^41^ to avoid the partial decomposition of the hydroperoxide group by lipase.^42^ From the HPLC–MS analysis of the hydrolysate, we identified specific monoacylglycerols depending on the HpODE binding positions on the glycerol backbone (i.e., *m/z* 481.3 generated from 1,3-OO-2-(HpODE)-TG or *m/z* 379.3 generated from 1-(HpODE)-2,3-OO-TG) (SI 2). Based on these results, we determined the identities of the 12 OO-HpODE-TG references, and further used the references for MS/MS and HPLC–MS/MS analysis.

### MS/MS and HPLC–MS/MS analysis of OO-HpODE-TG isomers

**Fig. 2:**
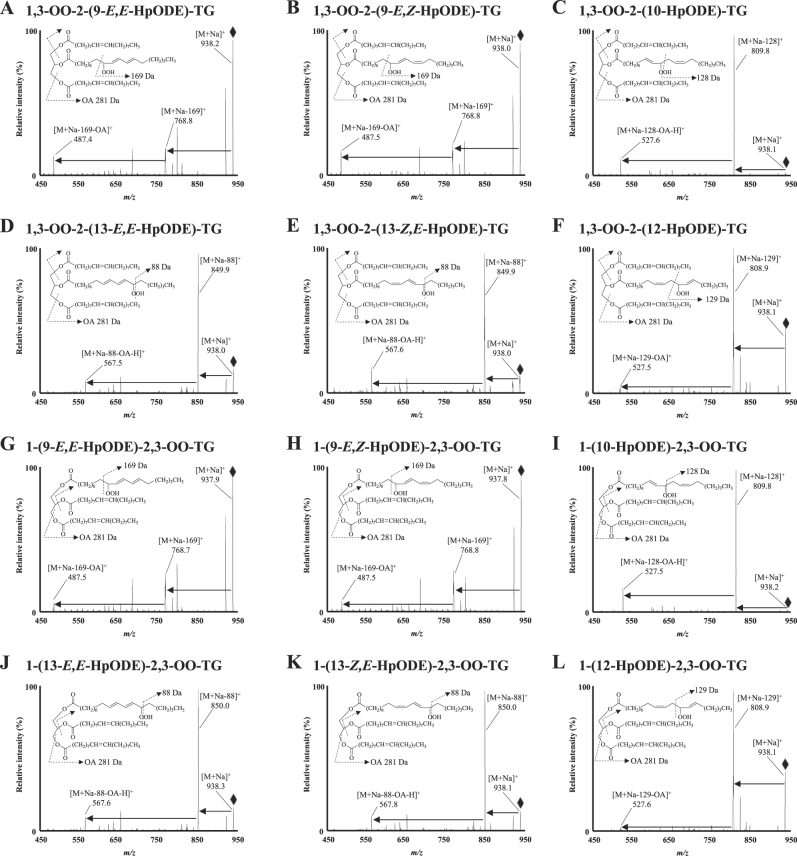
The product ion mass spectra of reference OO-HpODE-TG isomers (**A–L**). Reference OO-HpODE-TG isomers were dissolved in methanol containing 0.1 mM sodium acetate (1 µM) and infused directly into the MS/MS system at a flow rate of 10 μL/min. Insets show the speculated fragmentation patterns of OO-HpODE-TG isomers

As described in the Introduction, the isomerism of HpODE (i.e., hydroperoxide positional and cis-trans structural isomers) is essential to evaluate oxidation mechanisms in edible oil.^1,2,31,33,34,37^ In this study, we aimed to discriminate these isomers using MS/MS and HPLC separation techniques. Because the selective analysis of HpODE isomers were achieved in the presence of sodium in previous studies,^31–37^ OO-HpODE-TG references were dissolved in solvents containing sodium acetate. The references were analyzed by ESI operated in the positive ion mode. In each Q1 mass spectrum of the synthesized OO-HpODE-TG isomers, *m/z* 938.2 corresponding to [M+Na]^+^ was clearly detected (SI 3). Product ion analysis of these *m/z* 938.2 each gave a unique neutral loss (i.e., 169 Da for isomers bearing 9-HpODE (Fig. 2a, b, g and h), 128 Da for isomers bearing 10-HpODE (Fig. 2c, i), 129 Da for isomers bearing 12-HpODE (Fig. 2f, l) and 88 Da for isomers bearing 13-HpODE (Fig. 2d, e, j and k)). No significant difference in the product ion mass spectrum was observed between the OO-HpODE-TG geometrical isomers. Multiple reaction monitoring (MRM) pairs for HPLC–MS/MS analysis were selected based on the observed product ions (i.e., 938.0 > 768.8 for isomers bearing 9-HpODE, 938.0 > 809.8 for isomers bearing 10-HpODE, 938.0 > 808.8 for isomers bearing 12-HpODE and 938.0 > 849.8 for isomers bearing 13-HpODE).

**Fig. 3:**
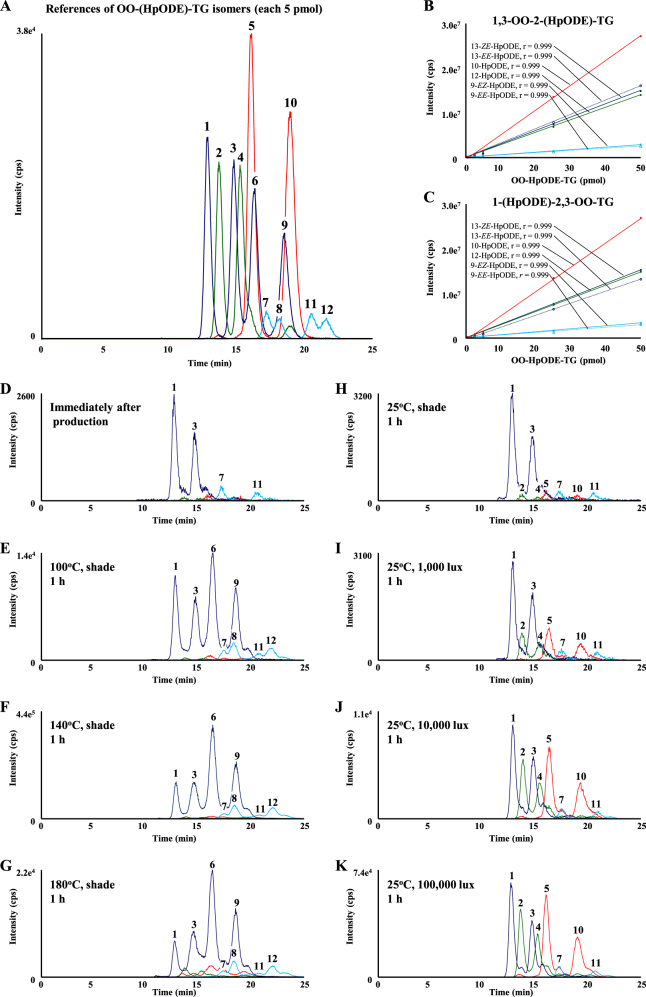
MRM chromatograms of reference OO-HpODE-TG isomers (**a**). OO-HpODE-TG isomer references (5 pmol each) were analyzed with MRM (*m/z* 938.0 > 768.8 for isomers bearing 9-HpODE, *m/z* 938.0 > 809.8 for isomers bearing 10-HpODE, *m/z* 938.0 > 808.8 for isomers bearing 12-HpODE and *m/z* 938.0 > 849.8 for isomers bearing 13-HpODE). Peaks 1–12 and their retention time were as follows: (1) 1,3-OO-2-(13-*ZE*-HpODE)-TG (12.6 min); (2) 1,3-OO-2-(12-HpODE)-TG (13.4 min); (3) 1-(13-*ZE*-HpODE)-2,3-OO-TG (14.5 min); (4) 1-(12-HpODE)-2,3-OO-TG (15.0 min); (5) 1,3-OO-2-(10-HpODE)-TG (15.9 min); (6) 1,3-OO-2-(13-*EE*-HpODE)-TG (16.1 min); (7) 1,3-OO-2-(9-*EZ*-HpODE)-TG (17.0 min); (8) 1,3-OO-2-(9-*EE*-HpODE)-TG (18.0 min); (9) 1-(13-*EE*-HpODE)-2,3-OO-TG (18.4 min); (10) 1-(10-HpODE)-2,3-OO-TG (18.8 min); (11) 1-(9-*EZ*-HpODE)-2,3-OO-TG (20.5 min); (12) 1-(9-*EE*-HpODE)-2,3-OO-TG (21.6 min). Calibration curves of reference OO-HpODE-TG isomers (1,3-OO-2-(HpODE)-TG (**b**) and 1-(HpODE)-2,3-OO-TG (**c**)). Different amounts of OO-HpODE-TG isomers (0.25–50 pmol) were analyzed by optimized LC–MS/MS MRM. MRM chromatograms of thermal- (**d–g**) and photo- (**h–k**) oxidized oil. Oxidized oils were 100-fold diluted with hexane, and a portion (50 µL) was analyzed using LC–MS/MS MRM. Peak numbers refer to the OO-HpODE-TG isomers described above

Subsequently, the geometrical isomerism of OO-HpODE-TG isomers were analyzed by HPLC–MS/MS. In this study, we used silica-based normal phase- (NP-) HPLC for the separation of OO-HpODE-TG isomers, because NP-HPLC is generally effective for the recognition of the hydroperoxide group during the analysis of fatty acid hydroperoxides.^43^ On the other hand, the solvents that are used in mobile phases for NP-HPLC are inadequate for ESI, because of their low proton transfer potential. In order to overcome this problem, the HPLC eluent was mixed with a post-column solvent consisting of methanol/2-propanol (1:1, v/v) to promote ionization. With the use of these MS/MS and HPLC techniques, we analyzed the 12 OO-HpODE-TG references. Under optimized HPLC–MS/MS conditions, all isomers were clearly detected (Fig. 3a). The retention time for each isomer is shown in Fig. 3a. For all isomers, calibration curves demonstrated good linearity within the range of 0.25–50 pmol/injection (Fig. 3b, c).

### Analysis of thermal- or photo-oxidized canola oil and the determination of oil oxidation mechanisms

In this study, we analyzed fresh canola oil (i.e., canola oil collected immediately after production) and oxidized oil using authentic references and optimized HPLC–MS/MS conditions. Fresh canola oil was oxidized by either thermal- (25–180 °C, shade) or photo- (25 °C, 1000–100,000 lux: corresponding to office lighting—direct sunlight) oxidation for 2 h, assuming that edible oil would be oxidized under common use or storage.

**Fig. 4:**
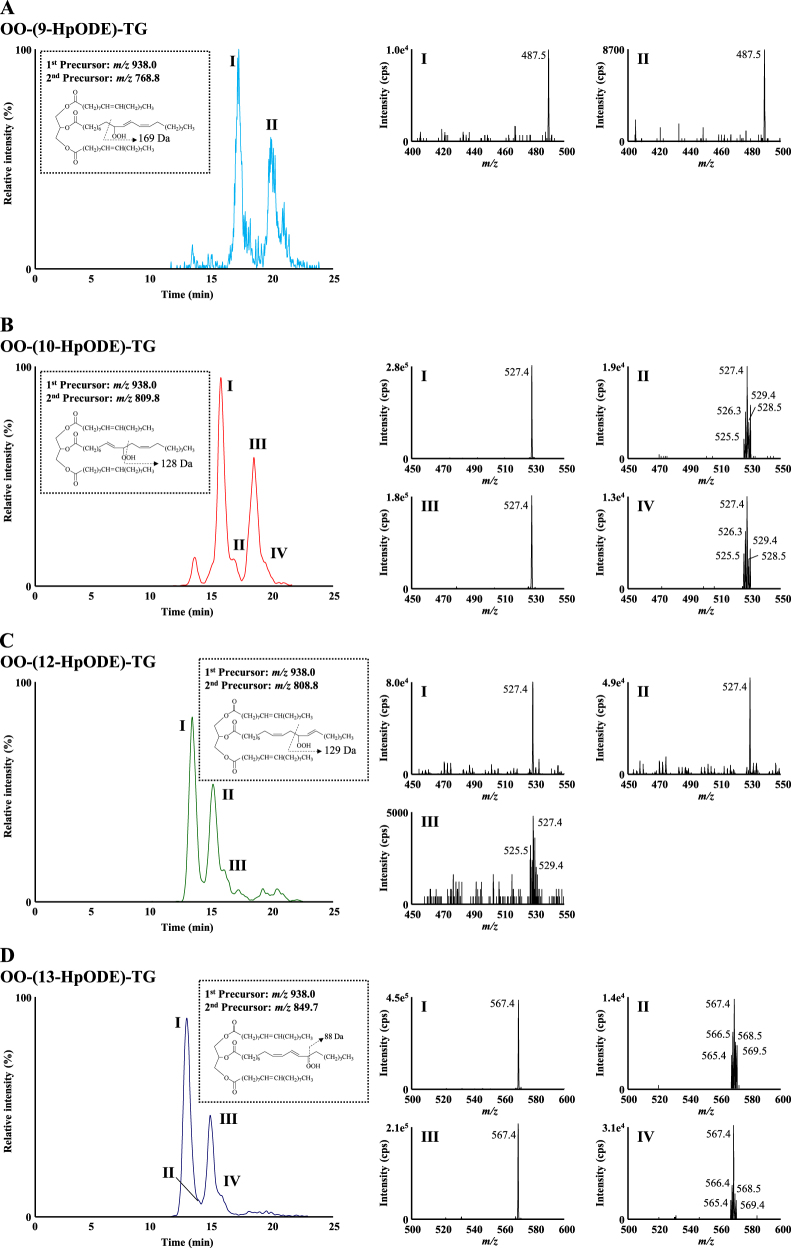
MS/MS/MS analysis of photo-oxidized oil. The ion *m/z* 938.0 was selected as a first precursor ion, and *m/z* 768.8 (OO-(9-HpODE)-TG (**a**); *m/z* 809.8 (OO-(10-HpODE)-TG (**b**); *m/z* 808.8 (OO-(12-HpODE)-TG (**c**); and *m/z* 849.7 (OO-(13-HpODE)-TG (**d**); were selected as a second precursor ion. Each chromatogram is a total ion current chromatogram of the product ion generated from the second precursor ion. MS/MS/MS spectra (I–IV) demonstrating product ions generated from the second precursor ion. Photo-oxidized oil were 100-fold diluted with hexane, and a portion (50 µL) was analyzed under optimized LC–MS/MS/MS conditions

The typical chromatograms of fresh, thermal-oxidized and photo-oxidized canola oil are shown in Fig. 3d–k. In the chromatograms of oxidized oil, some unidentified peaks were detected near the 1,3-OO-2-(HpODE)-TG and 1-(HpODE)-2,3-OO-TG. This suggests the possibility that other TGOOH molecular species were co-eluted with 1,3-OO-2-(HpODE)-TG and 1-(HpODE)-2,3-OO-TG. In this study, to secure highly selective analysis, HPLC–MS/MS/MS analysis was further performed (Fig. 4). For the MS/MS/MS analysis of OO-(9-HpODE)-TG, we used *m/z* 768.8 as a second precursor ion. As mentioned above, *m/z* 768.8 is generated from HpODE bearing hydroperoxide group at the ninth position (Fig. 2a, b, g and h). We then analyzed product ions generated from *m/z* 768.8, to check the other fatty acids type/combination (*sn*-1 and *sn*-3 or *sn*-2 and *sn*-3). Total ion current (TIC) chromatogram and mass spectra of the product ion generated from *m/z* 768.8 (photo-oxidized oil (10,000 lux)) are shown in Fig. 4a. On peaks I and II, fragmentation of *m/z* 768.8 predominantly generated the ion *m/z* 487.5 corresponding to the dissociation of an OA residue (281.3 Da). These results were also observed in the product ion mass spectra of OO-HpODE-TG isomers (Fig. 2). This result indicates that peaks I and II are composed of OO-(9-HpODE)-TG. Similarly, we analyzed OO-(10-HpODE)-TG in photo oxidized oil (10,000 lux) (Fig. 4b). On peaks I and III, fragmentation of *m/z* 809.8 predominantly generated the ion *m/z* 527.4 corresponding to the dissociation of the oleoyl residue and a proton (282 Da) (Figs. 2c, i and 4b). On the other hand, we found that peaks II and IV generated *m/z* 525.4 or 529.4 corresponding to the dissociation of a stealoyl or a linoleoyl residue, respectively. This suggests that peaks II and IV are isomers of OO-(9-HpODE)-TG (M.W. 915) such as stealoyl-linoleoyl-(13-HpODE)-TG (M.W. 915). The same MS/MS/MS analysis were performed on other isomers (Fig. 4c, d) as well as thermal-oxidized oil (Data not shown). These results prove that the targeted MRM peaks are definitely OO-(HpODE)-TG.

**Fig. 5:**
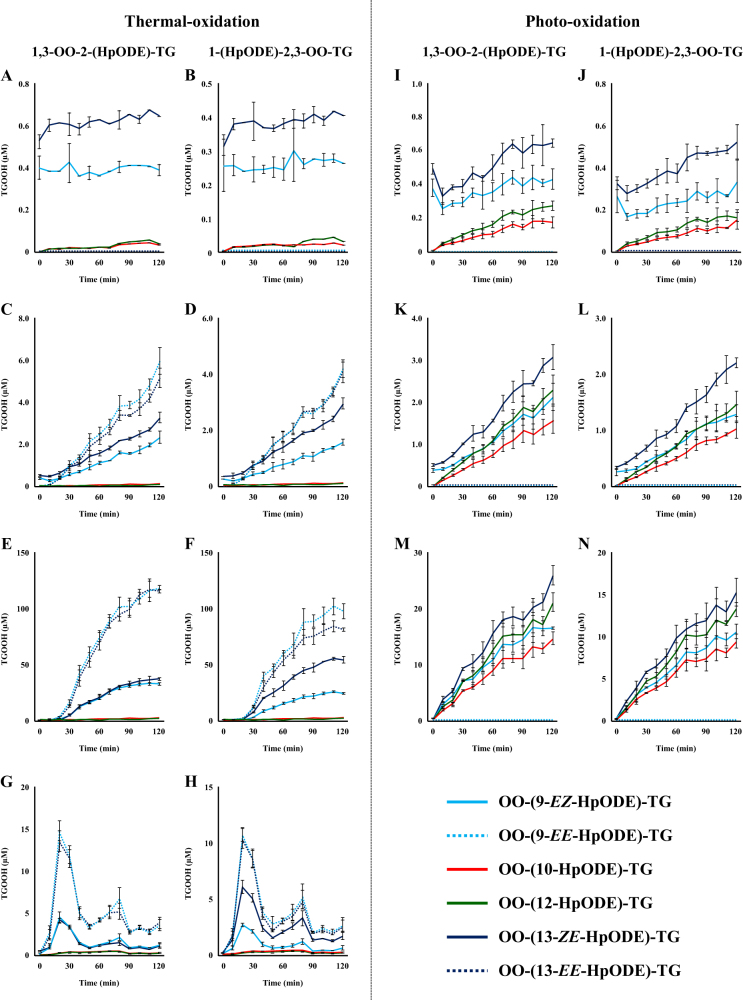
Concentrations of 1,3-OO-2-(HpODE)-TG and 1-(HpODE)-2,3-OO-TG isomers in oxidized oil. Fresh oil was oxidized under the following conditions: 25 °C, shade (**a**, **b**); 100 °C, shade (**c**, **d**); 140 °C, shade (**e**, **f**); 180 °C, shade (**g**, **h**); 25 °C, 1000 lux (**i**, **j**); 25 °C, 10,000 lux (**k**, **l**); 25 °C, 100,000 lux (**m**, **n**). Oxidized oils were 100-fold diluted with hexane, and a portion (50 µL) was analyzed under optimized LC–MS/MS MRM conditions

We then calculated the peak areas of OO-HpODE-TG isomers in fresh canola oil from MRM chromatograms. We found that OO-(9-*EZ*-HpODE)-TG and OO-(13-*ZE*-HpODE)-TG were present despite being analyzed immediately after production (Fig. 5, 0 min). When the oil was oxidized at 25 °C, OO-(13-*ZE*-HpODE)-TG tended to increase, while other OO-HpODE-TG isomers did not increase (Fig. 5a, b). On the other hand, when the oil was oxidized at high temperatures (100–180 °C), not only OO-(13-*ZE*-HpODE)-TG but also OO-(9-*EZ*-HpODE)-TG, OO-(13-*EE*-HpODE)-TG, and OO-(9-*EE*-HpODE)-TG significantly increased (Fig. 5c–h). Of these OO-HpODE-TG isomers, OO-(13-*EE*-HpODE)-TG and OO-(9-*EE*-HpODE)-TG were found to increase greater than OO-(13-*ZE*-HpODE)-TG and OO-(9-*EZ*-HpODE)-TG, even if TGOOH was decomposed by high temperature (180 °C). In contrast, in photo-oxidized oil, OO-(9-*EE*-HpODE)-TG and OO-(13-*EE*-HpODE)-TG were not detected at all illumination intensities (Fig. 5i–n). Also, it was found that OO-(12-HpODE)-TG and OO-(10-HpODE)-TG increased depending on the exposure time and illumination intensity. Detailed values and POV for each sample are shown in SI 4.

In the case of free fatty acid peroxidation, theoretically, 9-HpODE and 13-HpODE (or 10-HpODE and 12-HpODE) are equally generated by radical- or singlet oxygen-oxidation.^1^ However, in the present study concerning TG, the concentration of OO-(12-HpODE)-TG and OO-(13-*ZE*-HpODE)-TG were higher than that of OO-(10-HpODE)-TG and OO-(9-*EZ*-HpODE)-TG, respectively (Fig. 5). Moreover, in photo-oxidized oil, we found that the concentration of OO-(12-HpODE)-TG exceeded that of OO-(9-*EZ*-HpODE)-TG, despite the fact that OO-(9-*EZ*-HpODE)-TG is generated by both radical- and singlet oxygen-oxidation.^1^ These results suggest that steric hindrance is involved in TG peroxidation. Alam^29^ and Neff et al.^26^ reported that there are no *sn*-preference during the oxidation of trilinoleoyl-glycerol. On the other hand, other studies have reported that the fatty acid attached to the *sn*-2 position is tolerant to oxidation than the fatty acid attached to the *sn*-1 (*sn*-3) position.^44,45^ Further study would be required to elucidate steric hindrance, such as *sn*-position, fatty acid type and/or hydroperoxide position, during TG peroxidation.

In this study, we reliably identified OO-HpODE-TG molecular species/isomers peaks using MS/MS (MS/MS/MS) analysis and authentic references, although the analysis of TGOOH (even intact TG) is very difficult due to the various types of fatty acids and their combinations.^29^ The novel methods demonstrated that OO-(10-HpODE)-TG and OO-(12-HpODE)-TG were characteristically detected in photo-oxidized oil, and OO-(13-*EE*-HpODE)-TG and OO-(9-*EE*-HpODE)-TG increased depending on the temperature during thermal-oxidation. Our methods evaluated oil oxidation in levels that are unquantifiable with POV (even the oil oxidized by 1000 lux irradiation for 10 min).

### Determination of oxidation mechanisms of marketed canola oil

**Fig. 6:**
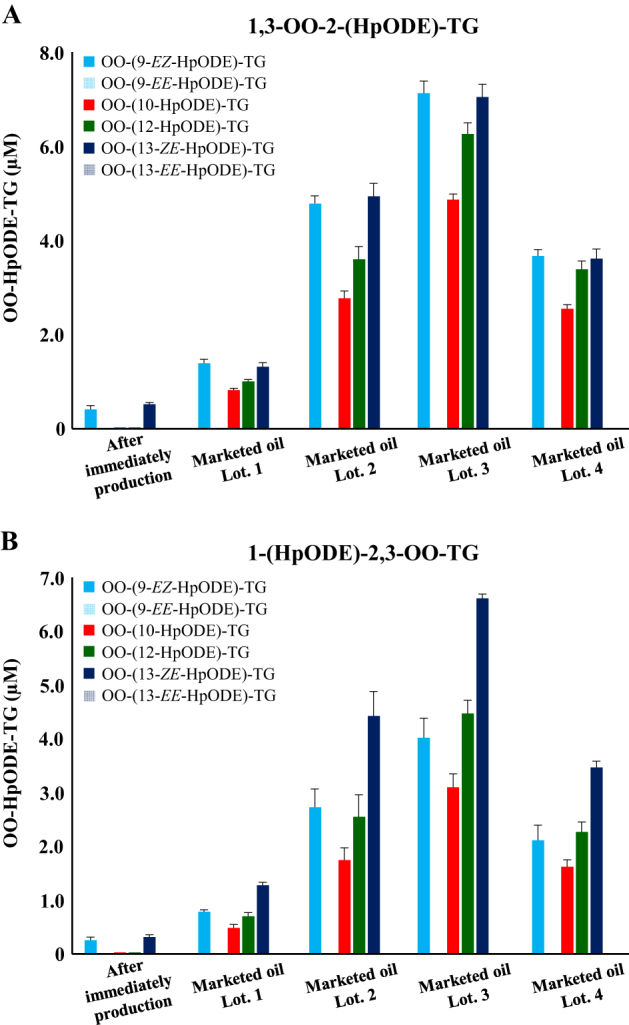
(**A** and **B**) Concentrations of OO-HpODE-TG isomers in marketed canola oil. Marketed oils were analyzed immediately after opening. Oils were 100-fold diluted with hexane and a portion (50 µL) was analyzed under optimized LC–MS/MS MRM conditions

Lastly, to evaluate the oxidation mechanisms during the transportation or storage of edible oils, we analyzed marketed canola oil products purchased from four different local retailers **(**Fig. 6**)**. The analyzed marketed canola oils were the same product as the fresh oil used above. The concentration of the total OO-HpODE-TG was 10–25 times higher in marketed oil compared to the fresh oil. Importantly, photo-oxidation products (i.e., OO-(12-HpODE)-TG and OO-(10-HpODE)-TG) were clearly detected in all marketed canola oils, although these isomers were not detected in the fresh oil. Furthermore, high temperature-oxidation products (i.e., OO-(13-*EE*-HpODE)-TG and OO-(9-*EE*-HpODE)-TG) were not detected in any canola oils. There was no difference in the isomer composition ratio between the marketed oils, despite the fact that the oil used in this study was purchased from different retailers. These results suggest that the oxidation of marketed canola oil is initiated by photo-oxidation around room temperature. Also, it can be assumed that canola oil is susceptible to photo-oxidation rather than radical oxidation. Generally, radical trapping reagents such as tocopherol is contained (and/or added) in commercial edible oils. Therefore, it can be assumed that radical oxidation is not enhanced at least before unsealing. On the other hand, since photo-oxidation products were accumulated in significant amounts, addition of singlet oxygen trapping reagents such as carotenoids^46–49^ and improvement of packaging techniques (e.g., use of brown bottle) may prolong the shelf life of edible oils.

Taken together, isomeric information of lipid hydroperoxides (i.e., geometrical and hydroperoxide-positional isomers) can be used to elucidate lipid oxidation mechanisms (thermal- and photo-oxidation). In this study, TGOOH isomers in canola oil were analyzed by preparing authentic isomer references and performing LC–MS/MS (MS/MS/MS) analysis. We demonstrated that the oxidation of marketed canola oil is predominantly caused by photo-oxidation. To the best of our knowledge, this is the first report that analyzed TGOOH at the molecular species and hydroperoxide positional-/geometrical-isomer levels. The method described in this paper would be valuable in the understanding of oil or food peroxidation mechanisms, and the development of preventive methods against food deterioration.

## Materials and methods

### Materials

Dioleoyl glycerol (mixture of 1,2-dioleoyl-3-hydroxy-*sn*-glycerol and 1,3-dioleoyl-2-hydroxy-*sn*-glycerol), LA, heptadecanoic acid, butyl hydroxy toluene (BHT), rose bengal, MxP, *N,N’*-dicyclohexylcarbodiimide (DCC) and dimethylaminopyridine (DMAP) were purchased from Wako Pure Chemical Industries, Ltd. (Osaka, Japan). Pyridinium *p*-toluenesulfonate (PPTS) was obtained from Sigma-Aldrich (St. Louis, MO, USA). Fresh canola oil was collected immediately after production at J-OIL MILLS, Inc. (Tokyo, Japan), and was stored under nitrogen gas and shading until use. Marketed canola oil products were purchased at four different local market in Sendai Japan. α-Lipase (Lipase F3G) was kindly provided from Amano Enzyme Inc. (Aichi, Japan). All other reagents were of the highest grade available.

### Determination of target TGOOH molecular species of canola oil

The fatty acid composition of TG in canola oil was analyzed by GC. Fresh canola oil (5 mg) was mixed with an internal standard (50 µg of heptadecanoic acid) in a screw cap test tube. Then, 2 mL of methanolic hydrogen chloride (5%, v/v) with 0.2 mL of benzene and 2 µL of BHT methanolic solution (0.01%, wt%) were added. The sample was methyl esterified at 100 °C under a nitrogen atmosphere. After 1 h, 5 mL of aqueous potassium carbonate (6%, wt%) and 1 mL of hexane were added. The mixture was partitioned by centrifugation (1000 × *g* for 5 min at 4 °C) into two layers. The upper hexane layer was collected, and remaining aqueous layer was re-extracted with 1 mL of hexane. The combined hexane layer was evaporated under a nitrogen gas stream, and the residue was dissolved in 1 mL of hexane. A 2 µL aliquot was analyzed with GC-4000 (GL Sciences Inc., Tokyo, Japan) equipped with a DB-225 column (length, 30 m; internal diameter, 0.32 mm; film thickness, 0.25 µm; Agilent Technologies, Santa Clara, CA, USA). Helium gas was used as the mobile phase. The injector and detector temperatures were set at 220 and 250 °C respectively. The gradient profile was as follows: 140–180 °C (8 °C/min linear), 180–220 °C (3 °C/min linear), and 220 °C (for 25 min).

TG molecular species were analyzed by Q1 mass- and product ion-scan mode using LC–MS/MS. A Prominence liquid chromatography system (Shimadzu, Kyoto, Japan) was equipped with a 4000 QTRAP mass spectrometer (SCIEX, Tokyo, Japan). Fresh canola oil was 10,000-fold diluted with methanol/2-propanol (100:1, v/v) and analyzed using an ODS column (5C_18_-MS-II, 5 µm, 4.6 × 250 mm, nacalai tesque, Kyoto, Japan) with a binary gradient consisting of solvent A (methanol containing 0.1 mM sodium acetate) and solvent B (2-propanol containing 0.1 mM sodium acetate). The gradient profile was as follows: 0–20 min, 50–70% B linear. The flow rate was 1.0 mL/min, and the column temperature was 40 °C. Elution was split at the post-column and one of the split eluents was sent to the MS/MS system at 0.2 mL/min. The MS parameters are shown in SI 5-Method 1 and 2. A 10 µL sample was injected into the LC–MS/MS system.

### Preparation of OO-(HpODE)-TG isomer references

#### Step I: Preparation of HpODE isomers

LA (2 g) was dissolved in 50 mL of methanol. Rose bengal (0.5 mg) was added, and the solution was exposed to light-emitting diode irradiation (50,000 lux, >30 °C) for 5 h. Rose bengal was removed by Sep-Pak Vac QMA (3 cc, 500 mg, Waters, MA, USA), and the solution was evaporated under a nitrogen gas stream. The residue was dissolved in 50 mL of hexane, and the sample (1 mL) was injected into a semipreparative HPLC system (Shimadzu, Kyoto, Japan) to isolate the HpODE isomers. Inertsil SIL-100A (5 μm, 10 × 250 mm, GL Sciences Inc., Tokyo, Japan) was eluted with hexane/2-propanol/acetic acid (100:1:0.1, v/v/v) at 20 mL/min.^43^ The column temperature was maintained at 40 °C, and the HpODE isomers were detected by UV absorbance at 210 nm. To refine the purity of the references, the obtained HpODE isomers were subjected once more to semipreparative HPLC under the same conditions.

#### Step II: Esterification of HpODE and diacylglycerol

The hydroperoxide group of the purified HpODE isomer references were protected with MxP (ref. 41). Briefly, each HpODE isomer was dissolved in acetonitrile (44 mM), to which 1.8 mM PPTS and 480 mM MxP were added. After 1 h, the reaction was stopped by adding an equivalent volume (to the reaction mixture) of water/methanol (4:1, v/v). The solution was loaded on a Sep-Pak Vac C18 (20 cc, 5 g, Waters, MA, USA) equilibrated with methanol/water (3:7, v/v). The column was washed with 40 mL of methanol/water (3:7, v/v), and the protected HpODE (HpODE-MxP) was eluted with 40 mL of methanol. The *cis-trans* isomers of 9-HpODE-MxP (i.e., 9-*EZ*-HpODE-MxP and 9-*EE*-HpODE-MxP), were separated by semipreparative RP-HPLC. An ODS column (Inertsil ODS-3, 10 µm, 20 × 250 mm, GL Sciences Inc., Tokyo, Japan) was eluted with methanol/water/acetic acid (10:2:0.01, v/v/v). The obtained six HpODE-MxP isomers (72 mM) were esterified with dioleoyl glycerol (78 mM) in chloroform containing DCC (74 mM) and DMAP (82 mM) under nitrogen gas stream for 1.5 h at 22 °C.^39^ The synthesized OO-(HpODE-MxP)-TG isomers were purified by semipreparative HPLC. Inertsil ODS-3 (10 µm, 20 × 250 mm) was eluted with methanol/2-propanol (3:2, v/v). The obtained OO-(HpODE-MxP)-TG isomers were deprotected as described previously.^41^ The resultant OO-HpODE-TG isomers (i.e., OO-(9-*EZ*-HpODE)-TG and OO-(9-*EE*-HpODE)-TG, OO-(10-HpODE)-TG, OO-(12-HpODE)-TG, OO-(13-*ZE*-HpODE)-TG and OO-(13-*EE*-HpODE)-TG) were chromatographically purified by semipreparative HPLC, using Inertsil ODS-3 (10 µm, 20 × 250 mm) eluted with methanol/2-propanol (3:2, v/v). OO-HpODE-TG *sn*-isomers was lastly separated with Inertsil SIL-100A (5 μm, 10 × 250 mm). Hexane/2-propanol (100:0.3, v/v) was used as mobile phase. The concentration of each reference was determined by measuring the concentration of OA, using the aforementioned GC method.

### Hydrolysis of OO-HpODE-TG references by α-lipase

The hydroperoxide group of each OO-HpODE-TG reference (45.7 µg) was protected with MxP as described above. The protected OO-HpODE-TG was dissolved in 0.1 mL of hexane. To the solution, 1 mL of α-lipase solution (10 mg/mL in 50 mM phosphate buffer (pH 6.8)) was added and the mixture was mixed vigorously at 37 °C for 10 min. The hydrolysate was extracted with 1 mL of hexane containing 0.1% (v/v) acetic acid, evaporated under nitrogen gas and dissolved in 500 µL of methanol. A portion of the sample (5 µL) was analyzed by LC–MS. The samples were analyzed using an ODS column (Inertsil SIL-100A, 5 μm, 2.1 × 100 mm, GL Sciences Inc., Tokyo, Japan) with a binary gradient consisting of solvent A (0.1 mM sodium acetate aqueous solution) and solvent B (methanol containing 0.1 mM sodium acetate). The gradient profile was as follows: 0–20 min, 50–70% B linear. The flow rate was 1.0 mL/min, and the column temperature was 40 °C. The MS parameters are shown in SI 5-Method 3.

### MS/MS and LC–MS/MS analysis of OO-HpODE-TG isomers

OO-HpODE-TG isomer references (1 µM) were dissolved in methanol/2-propanol (100:1, v/v) containing 0.1 mM sodium acetate. The samples were directly infused into 4000 QTRAP for Q1 mass- and product ion scan analysis. The MS/MS parameters are shown in SI 5-Method 4 and 5.

LC–MS/MS MRM analyses of oil samples were performed with Inertsil SIL-100A (5 μm, 4.6 × 250 mm, GL Sciences Inc., Tokyo, Japan). Hexane/2-propanol (100:0.3, v/v) was used as the mobile phase. A flow gradient was used as follows: 0–9 min; total flow 3–1 mL/min. The column was washed with methanol/2-propanol (1:1, v/v) for 1 h once per 10 injections. The column oven temperature was maintained at 40 °C. The column eluent was mixed with a post-column solvent consisting of methanol/2-propanol (1:1, v/v) containing 0.3 mM sodium acetate, at 0.4 mL/min. The combined flow was split at the post-column, and one of the split eluents was sent to the MS/MS system at 0.2 mL/min. OO-HpODE-TG isomers were detected using the MRM mode (SI 5-Method 6).

### Thermal- and photo- oxidation of canola oil

For thermal-oxidation, fresh canola oil (300 mL) was put into an amber 500 mL glass beaker, and oxidized under gentle stirring at 25, 100, 140 and 180 °C in the dark. For photo-oxidation, oil (300 mL) was put into a clear 500 mL glass beaker, and gently stirred under photo-irradiation (1000, 10,000 and 100,000 lux) at 25 °C. A portion of the oils (2 mL) were collected at 10 min intervals for 2 h (*n* = 3). The collected oils were 100-fold diluted with hexane, and a portion (50 µL) was analyzed using LC–MS/MS as described above in the MS/MS and LC–MS/MS analysis of OO/HpODE-TG isomers section.

### Peroxide value measurement

POV was measured according to the official method of the American Oil Chemists’ Society (AOCS) with slight modifications.^22^ Oxidized oil (1 g) was mixed with 5 mL of chloroform/acetic acid (2:3, v/v). To the mixture, saturated potassium iodide solution (0.1 mL) was added and gently mixed for 1 min. After the mixture was kept in the dark for 5 min, distilled water (5 mL) and 1% (wt%) starch solution (0.1 mL) were added. The mixture was centrifuged (1000×*g*, 5 min, 4 °C), and the lower layer was then removed. POV was calculated by titration with 10 mM sodium thiosulfate in units of 10 µL.

## Electronic supplementary material

Supplementaly Figure 1

Supplementaly Figure 2

Supplementaly Figure 3

Supplementaly Figure 4

Supplementaly Figure 5

## Electronic supplementary material

**Supplementary information** accompanies the paper on the *npj Science of Food* website (10.1038/s41538-017-0009-x).
