# Supplementary material for: Determination of triacylglycerol oxidation mechanisms in canola oil using liquid chromatography–tandem mass spectrometry
Source: NPJ Sci Food. 2018 Jan 12;2:1. doi: 10.1038/s41538-017-0009-x (PMC6550225; doi:10.1038/s41538-017-0009-x)
Supplement: Supplementary file 1 — Supplementaly Figure 1 [file 41538_2017_9_MOESM1_ESM.pptx]

## Slide 1
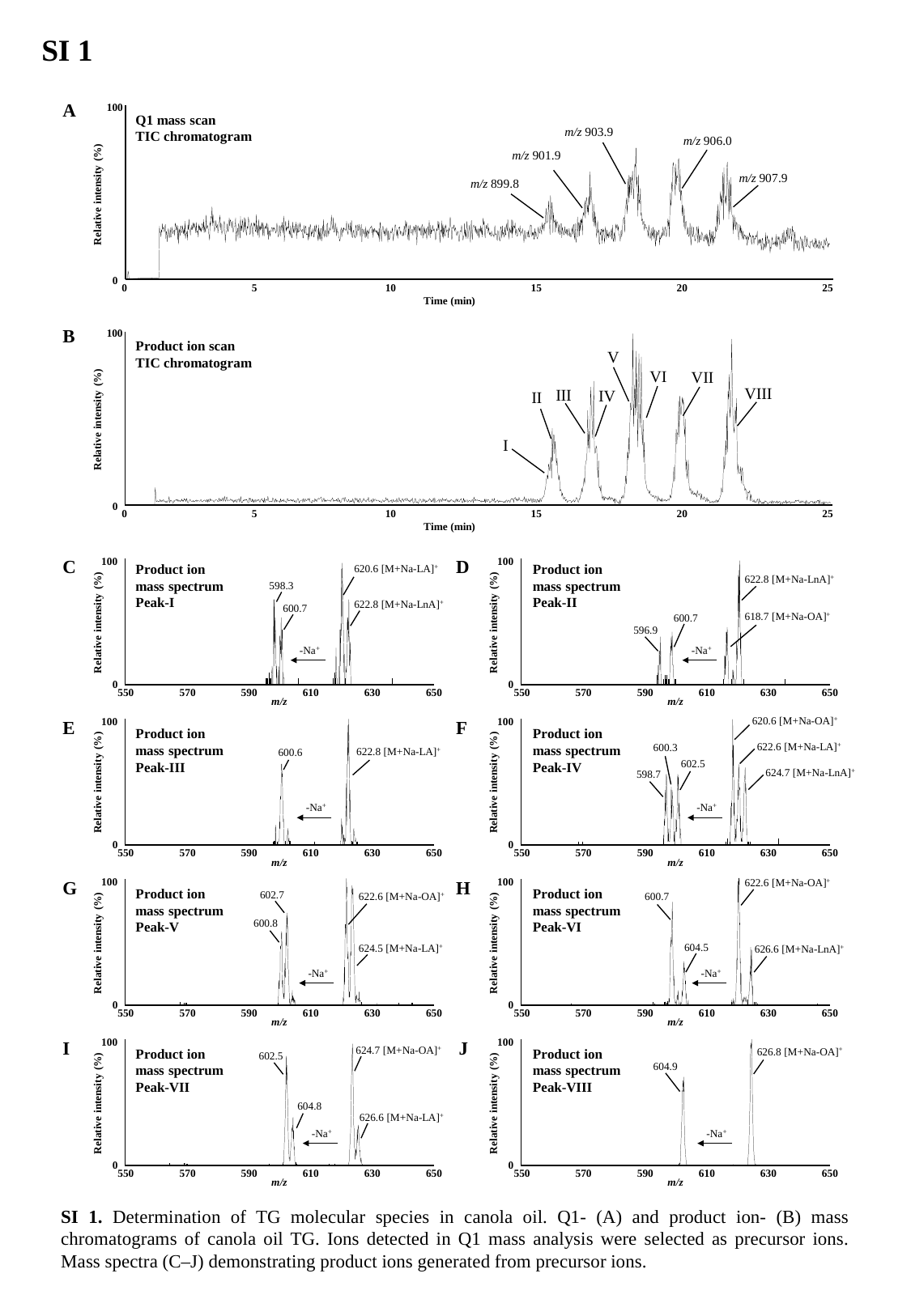

SI 1
SI 1. Determination of TG molecular species in canola oil. Q1- (A) and product ion- (B) mass chromatograms of canola oil TG. Ions detected in Q1 mass analysis were selected as precursor ions. Mass spectra (C–J) demonstrating product ions generated from precursor ions.
