# Supplementary material for: Determination of triacylglycerol oxidation mechanisms in canola oil using liquid chromatography–tandem mass spectrometry
Source: NPJ Sci Food. 2018 Jan 12;2:1. doi: 10.1038/s41538-017-0009-x (PMC6550225; doi:10.1038/s41538-017-0009-x)
Supplement: Supplementary file 2 — Supplementaly Figure 2 [file 41538_2017_9_MOESM2_ESM.pptx]

## Slide 1
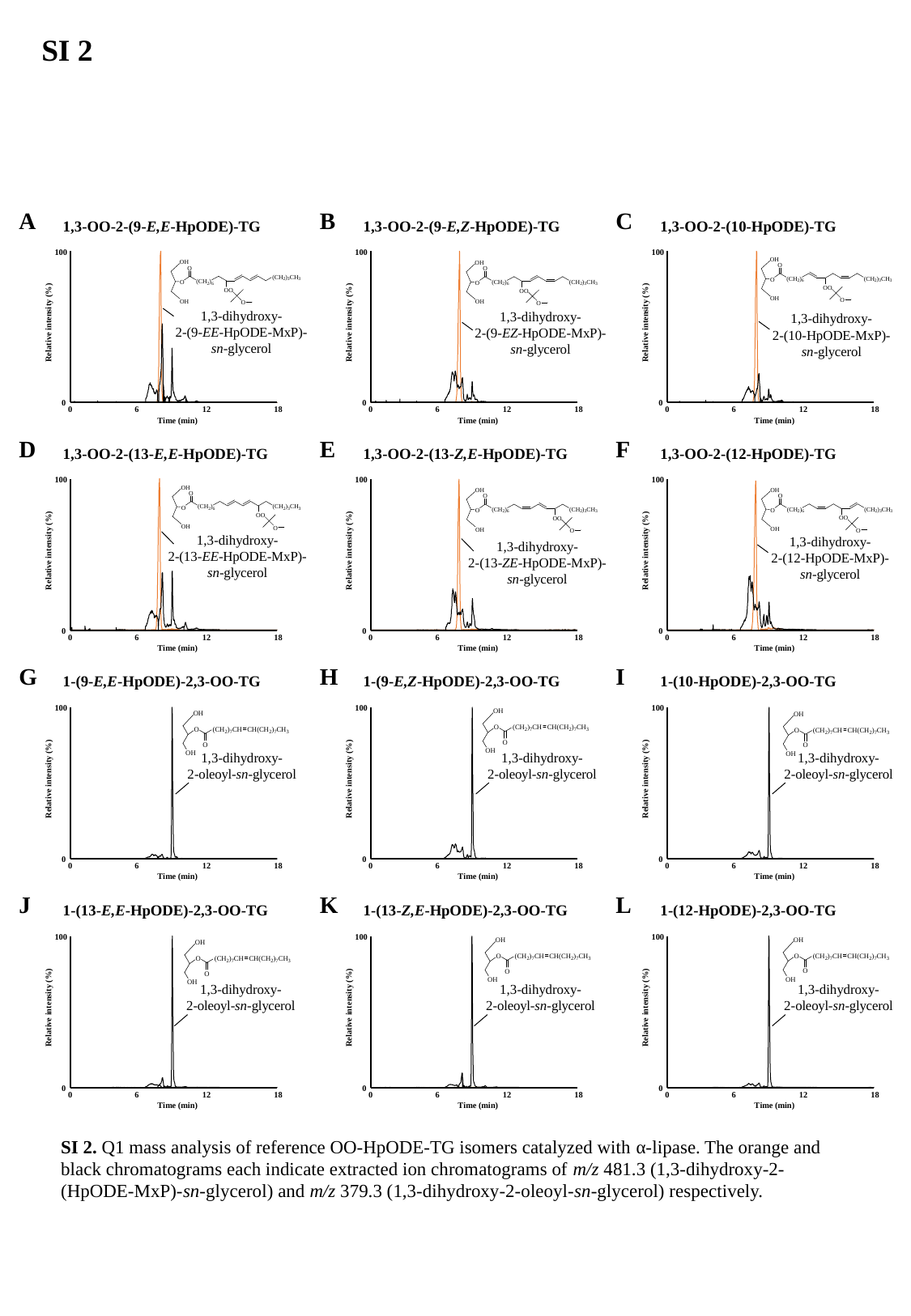

SI 2
SI 2. Q1 mass analysis of reference OO-HpODE-TG isomers catalyzed with α-lipase. The orange and black chromatograms each indicate extracted ion chromatograms of m/z 481.3 (1,3-dihydroxy-2-(HpODE-MxP)-sn-glycerol) and m/z 379.3 (1,3-dihydroxy-2-oleoyl-sn-glycerol) respectively.
