# Supplementary material for: Determination of triacylglycerol oxidation mechanisms in canola oil using liquid chromatography–tandem mass spectrometry
Source: NPJ Sci Food. 2018 Jan 12;2:1. doi: 10.1038/s41538-017-0009-x (PMC6550225; doi:10.1038/s41538-017-0009-x)
Supplement: Supplementary file 3 — Supplementaly Figure 3 [file 41538_2017_9_MOESM3_ESM.pptx]

## Slide 1
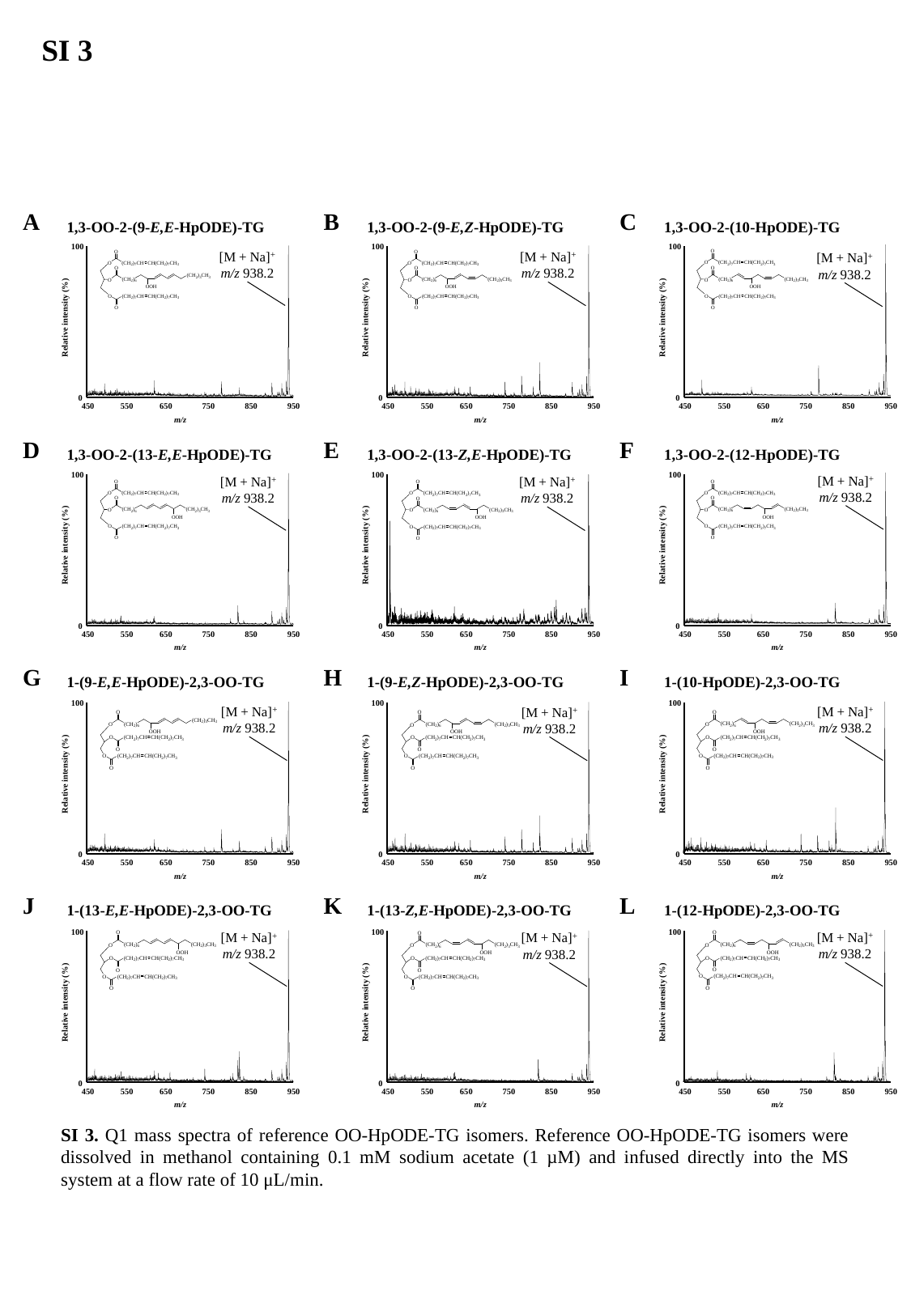

SI 3
SI 3. Q1 mass spectra of reference OO-HpODE-TG isomers. Reference OO-HpODE-TG isomers were dissolved in methanol containing 0.1 mM sodium acetate (1 µM) and infused directly into the MS system at a flow rate of 10 μL/min.
