# Supplementary figures and images for: Determination of triacylglycerol oxidation mechanisms in canola oil using liquid chromatography–tandem mass spectrometry
Source: NPJ Sci Food. 2018 Jan 12;2:1. doi: 10.1038/s41538-017-0009-x (PMC6550225; doi:10.1038/s41538-017-0009-x)

## Slide 1
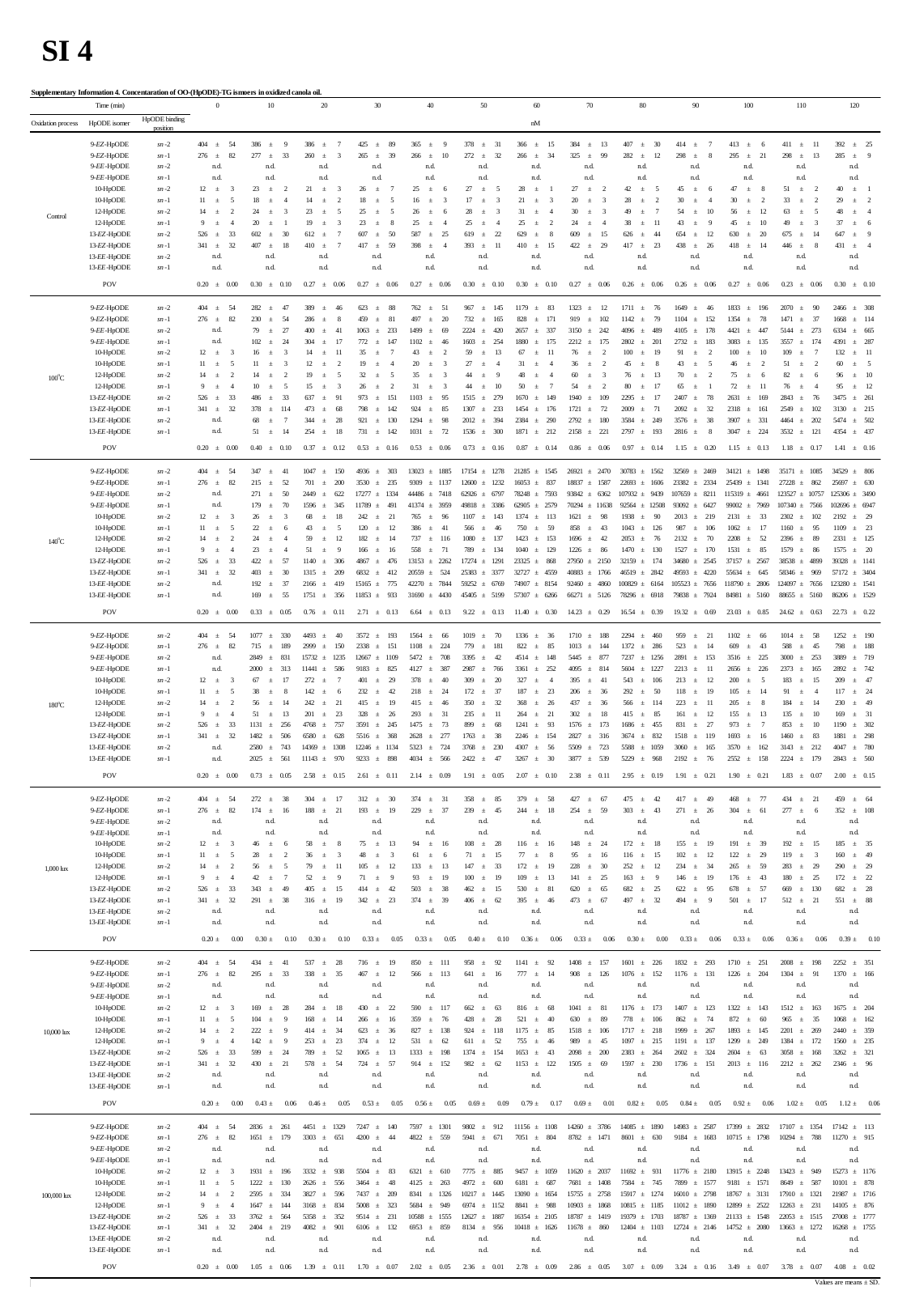

SI 4

Supplement: Supplementary file 4 — Supplementaly Figure 4 [file 41538_2017_9_MOESM4_ESM.pptx]

## Slide 1
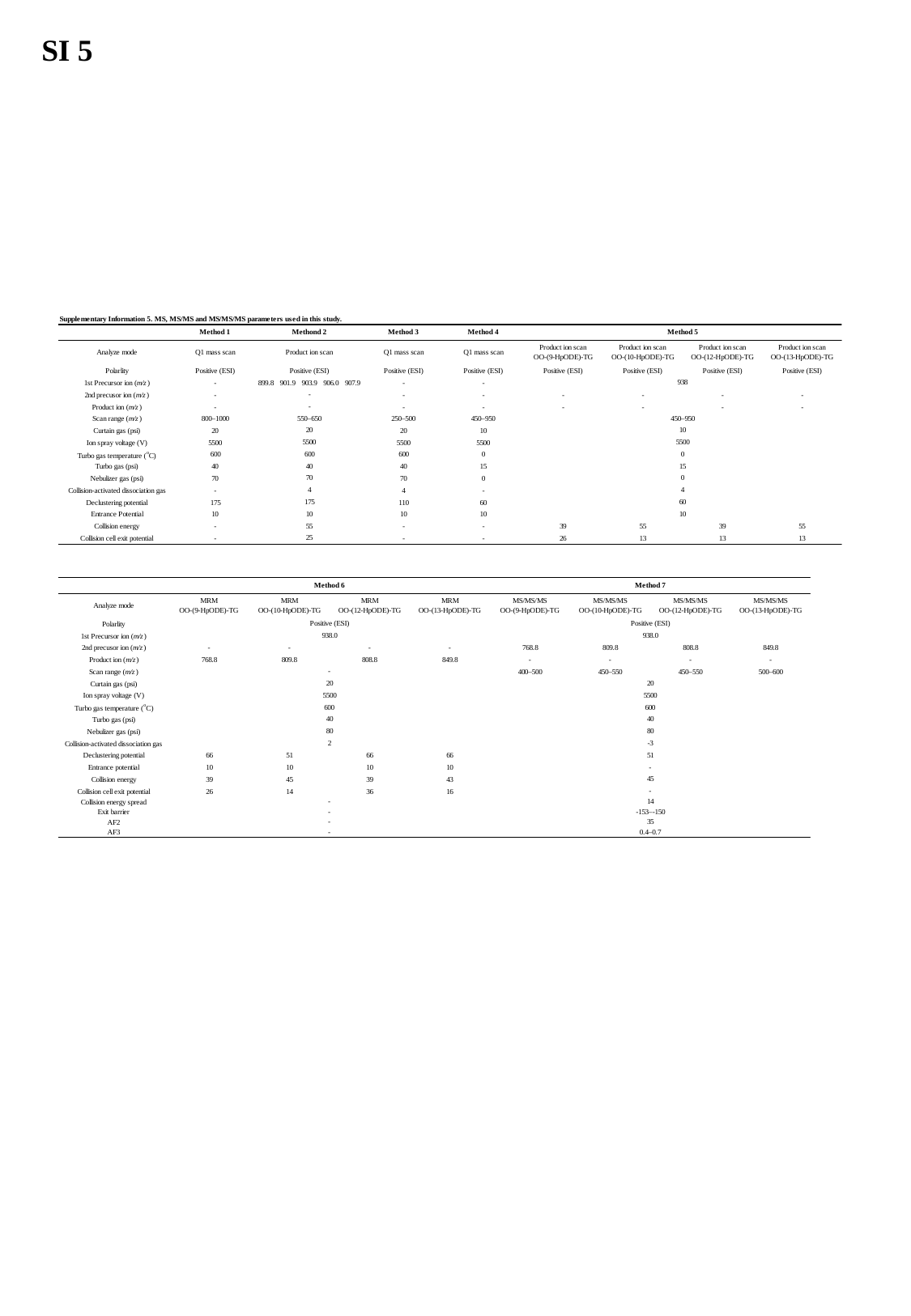

SI 5

Supplement: Supplementary file 5 — Supplementaly Figure 5 [file 41538_2017_9_MOESM5_ESM.pptx]
